# Nanotechnology: a promising method for oral cancer detection and diagnosis

**DOI:** 10.1186/s12951-018-0378-6

**Published:** 2018-06-11

**Authors:** Xiao-Jie Chen, Xue-Qiong Zhang, Qi Liu, Jing Zhang, Gang Zhou

**Affiliations:** 1 The State Key Laboratory Breeding Base of Basic Science of Stomatology (Hubei-MOST) and Key Laboratory of Oral Biomedicine Ministry of Education, School and Hospital of Stomatology, Wuhan University, Wuhan, 430079 People’s Republic of China; 2 School of Chemistry, Chemical Engineering and Life Sciences, Wuhan University of Technology, Wuhan, 430070 People’s Republic of China; 3 Division of Pharmacoengineering and Molecular Pharmaceutics and Center for Nanotechnology in Drug Delivery, Eshelman School of Pharmacy, University of North Carolina at Chapel Hill, Chapel Hill, NC 27599 USA; 4 Department of Oral Medicine, School and Hospital of Stomatology, Wuhan University, Wuhan, 430079 People’s Republic of China

**Keywords:** Oral cancer, Nanotechnology, Molecular imaging, Biomarker detection

## Abstract

Oral cancer is a common and aggressive cancer with high morbidity, mortality, and recurrence rate globally. Early detection is of utmost importance for cancer prevention and disease management. Currently, tissue biopsy remains the gold standard for oral cancer diagnosis, but it is invasive, which may cause patient discomfort. The application of traditional noninvasive methods-such as vital staining, exfoliative cytology, and molecular imaging-is limited by insufficient sensitivity and specificity. Thus, there is an urgent need for exploring noninvasive, highly sensitive, and specific diagnostic techniques. Nano detection systems are known as new emerging noninvasive strategies that bring the detection sensitivity of biomarkers to nano-scale. Moreover, compared to current imaging contrast agents, nanoparticles are more biocompatible, easier to synthesize, and able to target specific surface molecules. Nanoparticles generate localized surface plasmon resonances at near-infrared wavelengths, providing higher image contrast and resolution. Therefore, using nano-based techniques can help clinicians to detect and better monitor diseases during different phases of oral malignancy. Here, we review the progress of nanotechnology-based methods in oral cancer detection and diagnosis.

## Background

Cancer is a critical public health problem worldwide that has brought great burden to society. In 2016, an estimated 1,685,210 new cases and 595,690 cancer deaths occurred in the United States alone [1]. Oral cancer is the sixth most common cancer globally and has a 5-year survival rate of around 50% [2]. According to US cancer statistics, approximately 31,910 new cases of oral cancer and 6490 oral cancer deaths occurred in 2016 [3]. Oral cancer is an aggressive cancer that mainly affects oral epithelial cells, may develop metastasis, and even results in death [4]. The major type of malignancy is oral squamous cell carcinomas (OSCC), which accounts for more than 90% of all oral cancers [5]. These tumors may invade the mucosa of the tongue, buccal, floor of mouth, alveolar and the hard palate, and the tongue is reported to be the most common subsite, with poor prognosis [1, 6]. Oral carcinogenesis is often due to long-term exposure to various potential risk factors, which may lead to accumulation of multiple genetic mutations [4]. Several major risk factors for oral cancer, including smoking, alcohol consumption, and human papillomavirus infection, with smoking acting as the leading cause of cancer death [3, 7]. Besides, habitual use of the areca nut is another risk factor that closely associated with oral cancer, especially in Indian subcontinent [8].

The formation of oral cancer is a multifactorial and multistep process [6]. Oral leukoplakia, oral erythroplakia, oral lichen planus, oral submucous fibrosis, actinic keratosis, and discoid lupus erythematosus are common oral potentially malignant disorders (OPMD) that are known to have the potential for malignant transformation [8, 9]. Thus, early detection of OPMD and oral cancer is critical for the prognosis of diseases [5]. To date, scalpel biopsy and histopathological examinations are still the standard diagnostic procedures applied to ascertain the oral potentially malignant and malignant lesions [17, 18]. However, the biopsy procedure is often invasive, which may cause patients anxiety and discomfort [10]. The selection of resection margins depends largely on the histopathological assessments, and the results can be affected by the quality of the specimens and pathologists’ subjective judgments [11, 12]. In addition, the assessments are unable to detect small numbers of genetically abnormal cells at the margins, thus leaving the risk of recurrence [13, 14].

In the past few decades, a variety of pain-free diagnostic strategies have been developed. Non-invasive visual tools such as toluidine blue (TB) staining, autofluorescence (VELscope) and chemiluminescence (ViziLite) have been used solely or in combination as adjuvant tests to detect potentially malignant lesions [15–19]. In oral epithelial dysplasia cases, the sensitivity and specificity of TB, VELscope and ViziLite are reported to be 84.1% and 15.3, 77.3 and 27.8, 56.8 and 65.8%, respectively [15]. Exfoliated cells, serum, and saliva are the most commonly used non-invasive samples for oral cancer detection since they are easily accessible, convenient, and cost-effective [11, 20]. For oral cancer diagnosis, the sensitivity and specificity of exfoliative cytology is reported to be 93.5 and 50.6%, respectively [21]. The biomarker with high sensitivity and specificity in serum is combined detection of Cyclin D1 and epidermal growth factor receptor (EGFR), while the reliable marker in saliva is CD44 [22, 23]. Imaging techniques are used as diagnostic adjuncts to the histopathological assessments since they are noninvasive and done in real-time [24]. Radiographic imaging modalities-including magnetic resonance imaging (MRI), computed tomography (CT), cone beam computed tomography (CBCT), and positron emission tomography (PET)-are commonly used for clinical establishment of oral cancer stages and treatment plans [24, 25]. Raman spectroscopy, elastic scattering spectroscopy, diffuse reflectance spectroscopy, narrow-band imaging, and confocal reflectance microscopy are common optical diagnostic methods that distinguish malignant lesions from normal oral mucosa by reflecting changes within tissues through returned optical signals [11, 26–32].

However, these noninvasive methods still have some limitations [12]. The visual tools are highly subjective and depend on the expertise of the investigators [16–18]. The main deficiency of exfoliative cytology technology, which is based on the quantitative cytomorphometry and DNA aneuploidy, is the low detection specificity, resulting from the collection of disaggregated cells [12, 33, 34]. Moreover, the sensitivity for traditional detection methods is limited as the biomarkers with low concentrations in the tissue samples or body fluids may not be detected [35]. Although the imaging methods have provided real-time cancer cell morphology, their sensitivity for detecting small, earlier intraepithelial lesions are insufficient [36]. Thus, novel detection methods need to be explored to bring clinical benefits, including (1) accurately predicting the malignant risk of OPMDs, (2) specifically detecting oral cancer based on molecular targeting, (3) providing ultrasensitive detection strategies at nano-scale, (4) making real-time suggestions for the extent of surgical resection margins, and (5) monitoring oral cancer prognosis in a convenient way after treatment.

**Fig. 1:**
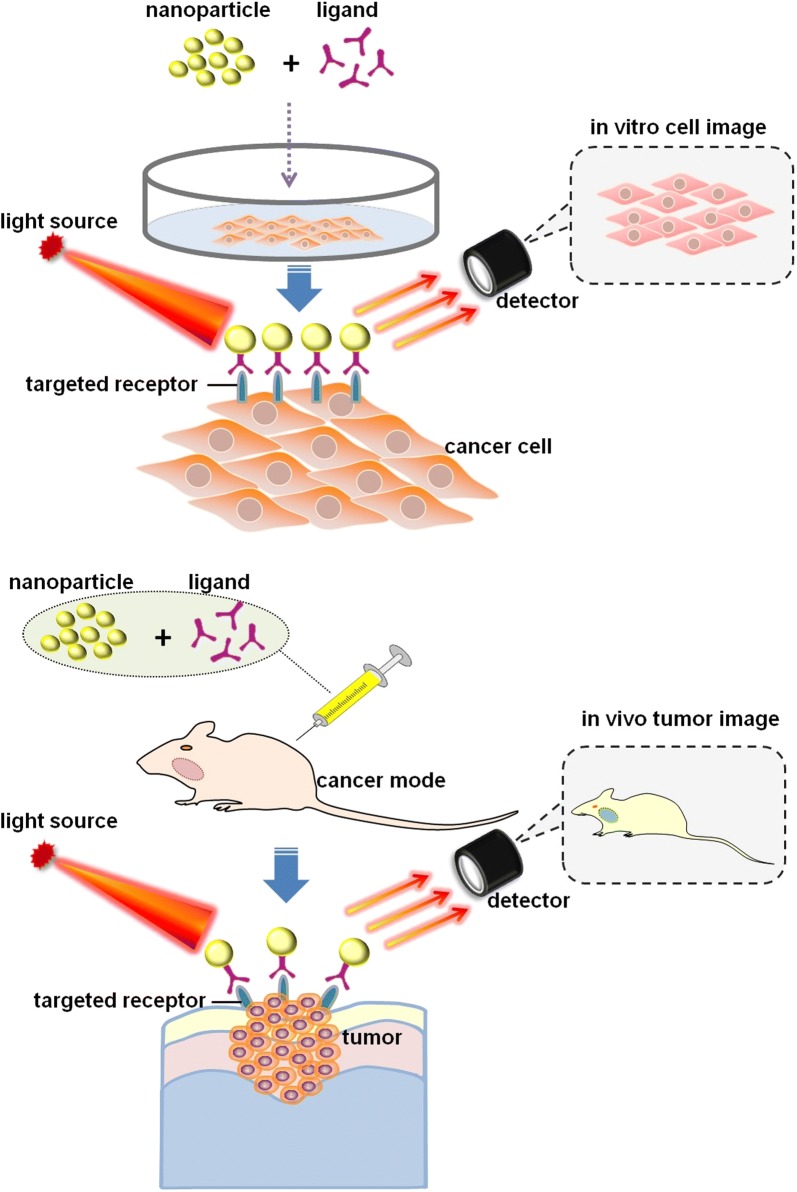
The application of nanotechnology for in vitro and in vivo bioimaging of oral cancer

According to the US National Nanotechnology Initiative, *nanotechnology* refers to the manipulation of matter with the length scale of 1–100 nm in at least one dimension [37, 38]. In the past few decades, nanotechnologies have been applied in various fields, especially in the medical field [39]. One of the most hotly researched subfield of nanotechnology is nanomedicine, which increases the possibility of specific targeted cancer therapy [40]. Moreover, nanotechnology is also a useful tool for cancer detection, and monitoring the disease as it metastasizes [41–44]. To date, nanotechnology has been applied in the detection and diagnosis of various cancers, such as cervical cancer, lung cancer, breast cancer, gastric cancer, nasopharyngeal cancer, and oral cancer [45–52]. As far as we know, the application of nano-based detection methods for oral cancer has not been systematically reviewed. In this review, we highlighted the various nanotechnologies that have been developed for oral cancer detection and diagnosis. The application of nanotechnology for in vitro and in vivo bioimaging of oral cancer was shown in Fig. 1.

## Nanotechnology-based detection and diagnostic methods

### Nano-based molecular imaging

#### Magnetic resonance imaging

Magnetic resonance imaging (MRI) is reported to be suitable for the assessment of the primary tumor and bone invasion, as well as the outlining of the actual tumor borders during surgery [25, 53]. Commonly used positive MRI contrast agents-Gd3+ complexed with diethyltriamine-pentaacetic acid (Gd-DTPA) or tetra azacyclododecane-1,4,7,10-tetraacetic acid (Gd-DOTA)-can shorten tissue longitudinal relaxation times (T1) [54]. However, the contrast agents distribute throughout the entire body after being intravenously injected, but do not specifically accumulate in tumors. In addition, the blood circulation life time for Gd-DTPA or Gd-DOTA is very short, approximately only 1–1.5 h [55]. The contrast agents usually consist of superparamagnetic nanoparticles with coating layers [56].

With the advancement in nanotechnology, various types of nanoparticles have been applied as specific MRI contrast agents for cancer screening [54]. Nano-contrast agents have the ability to recognize unique cell surface markers and prolonged blood circulation half-life, exhibiting better MRI contrast properties [57]. The most commonly studied superparamagnetic iron oxide (SPIO) and ultrasmall superparamagnetic iron oxide (USPIOs) nanoparticles, which can shorten T2 and T2*, have already been used as negative contrast agents for detecting liver and spleen diseases [58].

**Fig. 2:**
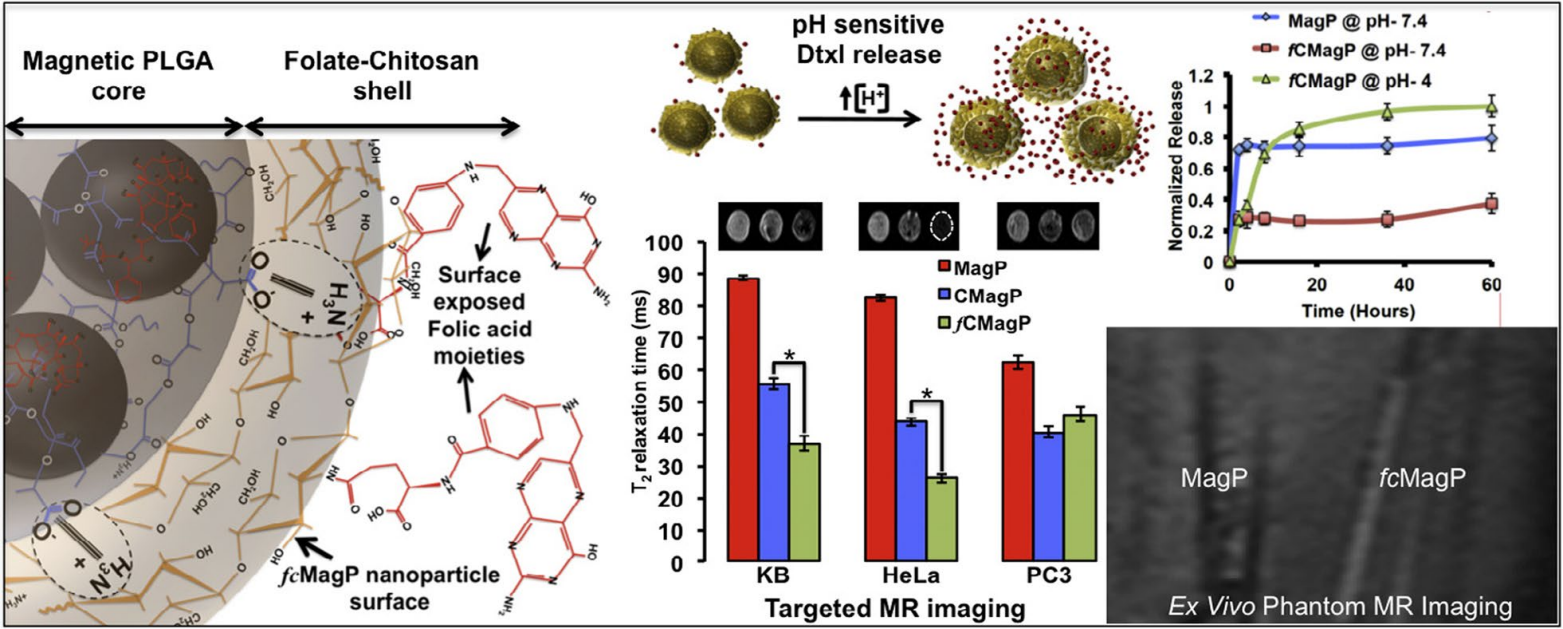
Representation of the magnetic core–shell hybrid nanoparticles for receptor targeted MRI (Reprinted with permission from [59]. Copyright 2017 Journal of Colloid and Interface Science)

Nano-contrast agents have also been studied in oral cancers. For example, Asifkhan et al. combined the folate preconjugated chitosan and magnetic poly (lactide-*co*-glycolide) (PLGA) nanoparticles to create an MRI contrast agent (Fig. 2) [59]. The overall T2 relaxation time was shortened, and the nanoparticle relaxivity was enhanced thereby providing better imaging contrast [59]. Meanwhile, the folate receptor positive KB oral cancer cells showed increased nanoparticle uptake and caused significant enhancement in cytotoxicity [59]. This nano agent not only provided high contrast cancer imaging but also simultaneously provided cancer therapy. Another novel magnetic nano-contrast agent was developed based on Gd3+ doped amorphous TiO_2_ and was suitable for T1 weighted MRI [60]. The size of this agent was reported to be about 25 nm, which is much smaller than SPIO (50 nm) [58]. The potential of inducing hemolysis, platelet aggregation, and plasma coagulation was studied, and no adverse reaction was reported [60]. As a consequent, the folic acid conjugated nanoparticles were specifically aggregated on the surface of folate receptor positive oral cancer KB cells, leaving normal L929 cells unstained [60]. Notably, this nano-contrast agent showed enhanced longitudinal relaxivity, magnetic resonance, and excellent biocompatibility for MRI.

#### Optical coherence tomography

Optical coherence tomography (OCT) is a direct simulation of ultrasound. It produces cross-sectional architectural images of subsurface tissues, such as epithelial layers and basement membranes, using infrared light with a penetration depth of about 2 mm, and is suitable for early oral cancer detection and oral dysplasia monitoring [61]. The resolution of OCT is reported to be around 10 μm which is higher than that of other noninvasive diagnostic techniques, such as CT, MRI, and ultrasound [50, 62]. Although OCT is a non-invasive and real-time clinical diagnostic method for cell and stromal morphology imaging, the contrast remains insufficient, especially between neoplastic and normal tissues [63].

Gold nanoparticles are promising OCT contrast agents. They are biocompatible, easy to synthesize, and can provide localized surface plasmon resonances at near-infrared wavelengths that avoid predominant absorption in tissues [64]. For example, the EGFR monoclonal antibodies conjugated Au nanoparticles with a diameter of 71 nm have been applied to enhance the contrast of OCT images of oral dysplasia in a hamster model [65]. Meanwhile, microneedles and ultrasound were utilized to overcome the obstacle for Au NP delivery. This multimodal delivery was demonstrated to be effective in improving OCT penetration depth and resulted in an approximately 150% increased contrast level in oral carcinogenesis [65].

#### Photoacoustic imaging

Photoacoustic imaging is a new emerging optical diagnostic technology. By using a short laser pulse, it generates ultrasound transients from tissues, thereby causing transient thermoelastic expansions after optical absorption [66–68]. These photoacoustic waves are being then transformed into photoacoustic images according to their arrival times after collected by an ultrasound transducer [69, 70]. The ultrasound provides high spatial resolution for structural phenotyping and is a useful tool for assessing lymph nodes following a radical surgery [71, 72]. Consequently, the optical contrast can be significantly improved while maintaining the high spatial resolution of ultrasound [73]. Compared to conventional optical imaging, photoacoustic imaging has improved imaging depth, about 6 cm [69]. Though various exogenous contrast agents-such as methylene blue, ICG, and GNs-have been used to enhance the photoacoustic imaging contrast, the gold nanoparticles are considered a more attractive contrast agent due to their ability to conjugate biomolecules and their production of stronger photoacoustic imaging signals [67, 69, 74]. To date, photoacoustic imaging has demonstrated great potential in brain, breast, and prostate cancer diagnosis [67, 73, 75, 76].

**Fig. 3:**
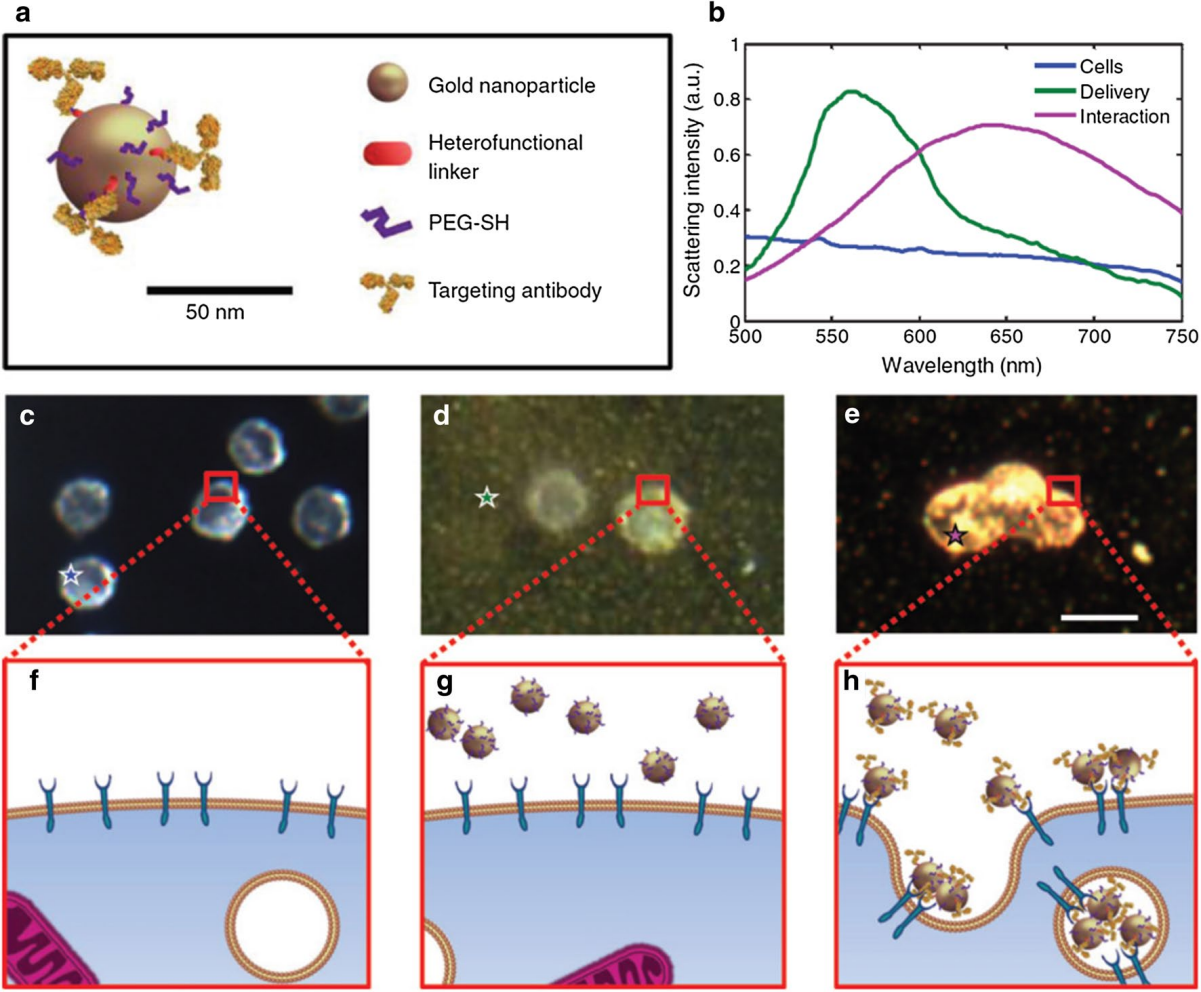
Representation of the photoacoustic imaging using anti-EGFR antibody conjugated molecularly activated MAPS. **a** A schematic of the EGFR-targeted MAPS; **b** optical spectra obtained hyperspectral dark-field microscopy; **c**, **f** cancer cells in the absence of gold nanoparticles; **d**, **g** cells in the presence of nonspecific AuNPs; **e**, **h** cells labeled with MAPS (Reprinted with permission from [77]. Copyright 2014 Cancer Research)

Luke et al. introduced ultrasound-guided spectroscopic photoacoustic imaging technology for detecting lymph node micrometastases in a metastatic murine model of OSCC (Fig. 3) [77]. Using anti-EGFR antibody conjugated molecularly activated plasmonic nanosensors (MAPS), the study showed that the MAPS shifted their absorption spectrum to the near-infrared region [77]. In addition, large ultrasound-guided spectroscopic photoacoustic signals appeared in micrometastases as small as 50 mm within 30 min after MAPS injection [77]. These findings offer an alternate to sentinel lymph node biopsy analysis of oral cancer resection.

#### Surface plasmon resonance scattering

Surface plasmon waves are formed by collective oscillation of conduction electrons in noble metals [78]. Recently, gold nanoparticles have been commonly applied for surface plasmon resonance scattering since they can resonantly scatter visible and near-infrared light due to their surface plasmon oscillation [78]. In addition, they are easy to prepare, readily bioconjugated, and have low cytotoxicity, making them suitable for biomolecular labeling and targeting [79]. It is reported that the conjugated nanoparticles tended to aggregate together, inducing a greatly enhanced surface plasmon resonance scattering compared to unconjugated nanoparticles [80].

El-Sayed et al. recorded surface plasmon resonance scattering images and surface plasmon resonance absorption spectra after cell incubation [81]. Light-scattering images showed that the EGFR conjugated nanoparticles bind specifically to the surface of the cancer cells with high concentration, while the binding to noncancerous cells was nonspecific and random [81]. Micro absorption spectra showed that the absorption maximum for conjugated nanoparticles was 545 nm, without aggregation tendency, while unconjugated colloidal gold nanoparticles accumulated inside cells and aggregated with an absorption maximum around 552 nm [81]. As a result, the anti-EGFR antibody conjugated nanoparticles showed 600% greater affinity to malignant oral epithelial cell lines HOC 313 clone 8 and HSC 3 than to the nonmalignant cell line HaCaT [81]. In addition, the surface plasmon resonance property of gold nanoparticles was shown to have the ability to increase Raman scattering in saliva samples of oral cancer patients [63, 78]. High optical signals were produced by enhanced surface plasmon resonance when the gold nanoparticles gathered around the target cancerous cells, due to their conjugation with anti-EGFR [63]. The sensitivity was observed to be around 70% of the current technique, which needs to be further improved [63].

#### Surface-enhanced Raman spectroscopy

Raman spectroscopy is a vibrational spectroscopic technique based on inelastic interactions between light and matter [82]. The normal, premalignant, or malignant lesions are distinguished by inelastic scattering of light, which can be a laser in the visible, near-infrared, or near-ultraviolet range [83]. The signals in normal tissues are homogeneous but heterogeneous in malignant cells, reflecting the changes in chemical characterization and molecular structure of the lesions [84]. Raman spectroscopy is a near-field effect and has a low penetration depth. Its clinical application has been limited by the weak Raman signal intensity and the slow speed of spectrum acquisitions [78, 83].

**Fig. 4:**
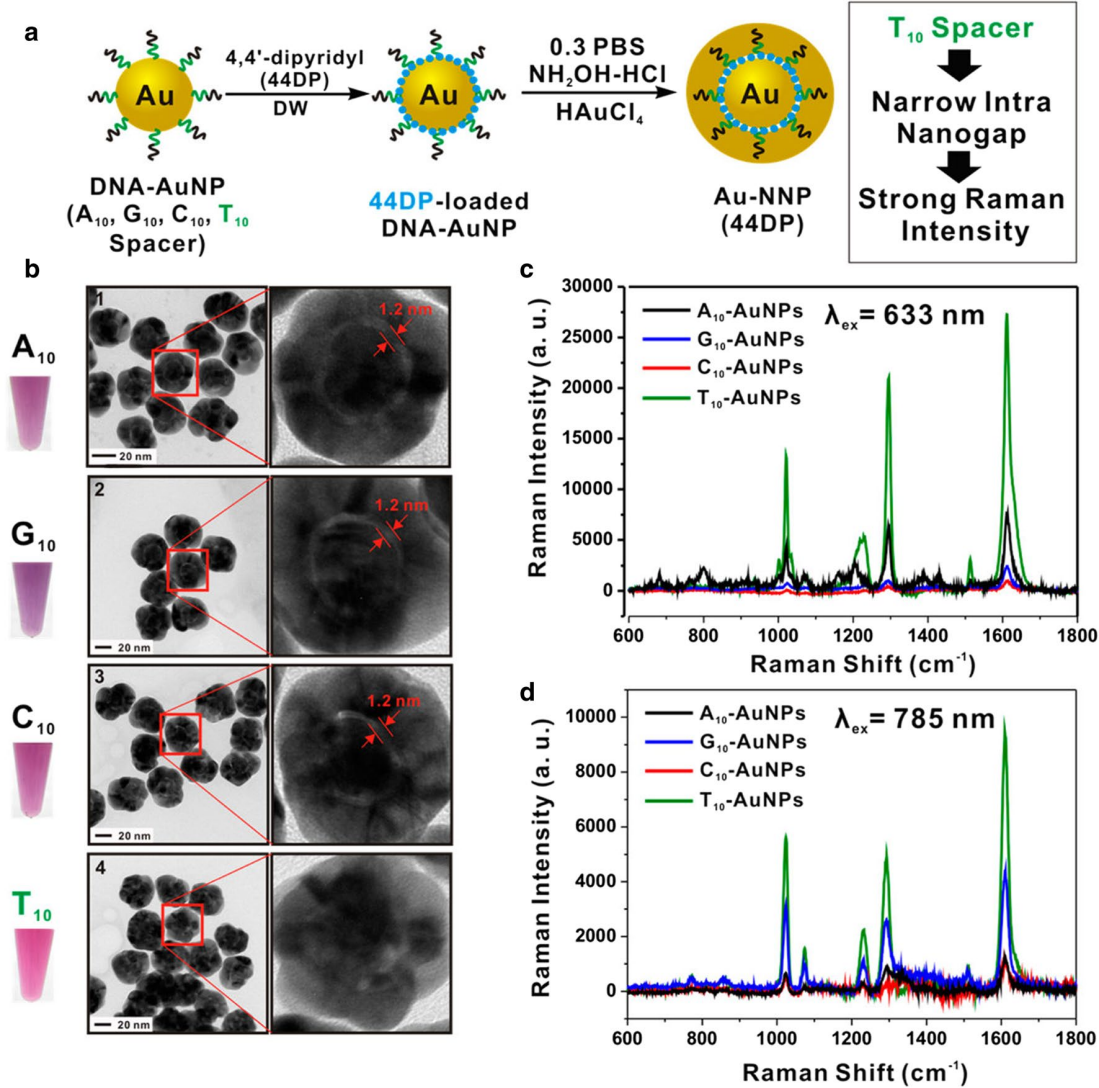
Graphical representation of the SERS active gold nanoparticles for oral cancer cell HSC-3 imaging. **a** synthetic scheme of Raman dye (44DP)-coded Au-NNPs using four different kinds of DNA-AuNPs as core particles. **b** the solution color and HR-TEM image of 44DP-coded Au-NNPs. **c**, **d** Raman spectra of 44DP-coded Au-NNP solution prepared from four different spacer DNA with an excitation of 633 (**c**) and 785 nm (**d**) (Reprinted with permission from [89]. Copyright 2015 Nano Letters)

Recently, nanoparticles have been applied as exogenous contrast agents, in order to acquire Raman signal with high speed and resolution [85–87]. After directly adsorbed on the nanoparticle surface, the molecules emit an amplified Raman scattering intensity, known as surface-enhanced Raman scattering (SERS) [83, 88]. A study introduced small, spherical, near-infrared region sensitive and SERS active gold nanoparticles with highly narrow intra-nanogap structures for single oral cancer cell HSC-3 imaging (Fig. 4) [89]. The gold nanoparticles can selectively target intracellular organelles and were specifically distributed in cytoplasm, mitochondria, and nuclei. Finally, high speed Raman imaging was achieved within 30 s with a high resolution of 50 × 50 pixels [89].

Nanospheres, nanorods, nanocubes, nanobranches, and nanobipyramids are different shapes of gold nanoparticles [90, 91]. Gold nanorods (GNRs) have received much attention for molecular imaging because of their advantage of higher index sensitivity over spherical and cubic gold nanoparticles, which means minor changes in the surrounding environment of GNRs can result in significant longitudinal surface plasmon resonance (LSPR) peak wavelength variation [90, 92]. Since the index sensitivities and longitudinal plasmon wavelengths of nanorods increase with aspect ratios, the use of nanorods with large aspect ratios can provide near-infrared region plasmon wavelengths and high index sensitivity for optical techniques [90, 91].

Wang et al. conjugated GNRs with rose bengal (RB), a specific probe for oral cancer cell target, and monitored optical absorption in the near-infrared region [93]. The RB molecules have the ability to bind with the protein or nucleic acid of cancer cell lysate, whereafter the RB-GNR probes aggregated, inducing red-shift in the near-infrared absorption wavelength [93]. This RB-GNR platform provided a specific and quantitative method for oral cancer cell lysate analysis with a detection sensitivity of 2000 cells/ml [93]. Liu et al. described a paper-based SERS technology in combination with exfoliative cytology for screening of exfoliated cells from oral cancer patients and healthy individuals [94]. Cells were placed on a plasmonic paper with GNRs adsorbed on it, and spectra were acquired afterward. Sensitivity and specificity were both 100% for distinguishing exfoliated cells from normal and cancer tissues, based on the I_1600/1440_ and I_1440/1340_ peak ratios of the spectra values [94]. This paper-based SERS platform has overcome the drawbacks of traditional exfoliative cytology, such as low sensitivity and subjective cytologic interpretation [94].

#### Diffusion reflection imaging

In diffusion reflection imaging, a small portion of the white light entering the tissue is absorbed or transmitted, while the rest undergoes multiple elastic scattering and gets diffusely reflected [95]. The reflected light is greatly affected by cytologic and morphologic changes during epithelial tissue cancerization, including nuclear size, collagen content, extracellular matrix structure, epithelial thickness, and blood flow variation [28, 96]. It is reported that recording diffuse reflectance images can help to determine surgical margins and is a useful tool to differentiate normal mucosa, OPMD, and oral cancer [96–98].

In oral cancer, 14.3% of tumor margins after surgical excision were identified to have residual carcinoma [99]. Accurate determination of tumor margins is critical for complete surgical resection of residual diseases in oral cancer and may reduce the high rate of recurrence [100]. The accuracy of routine microscopic examination after frozen sections is limited by the 30.7–47.3% shrinkage of the frozen tissues [101]. Meanwhile, for the paraffin-embedded tissue section, results are only available after the operation, making the intraoperative identification challenging [101]. Thus, efforts should be made to achieve a real-time and high sensitive way for more complete tumor resections.

Ankri et al. conjugated GNRs to monoclonal antibodies against EGFR and evaluated the margins of human OSCC specimens by diffusion reflection imaging [102]. Air scanning electron microscopy was used to visualize the nanorods in tissues, showing the GNRs-EGFR spread a distance of 1 mm between the tumor and the healthy regions. Diffusion reflection imaging was then performed in a resolution of 1 mm, suggesting that the tumor edge is in the region of 4–5 mm, which is consistent with the commonly used cutoff of 5 mm for a close margin [100]. This study group has also tested diffusion reflection imaging of GNRs-EGFR on a mice OSCC model induced by 4-nitroquinoline-*N*-oxide [103]. GNRs specifically attached to areas histologically identified as OSCC, with high reflectance at 780 nm over 17 intensity units. The overall specificity and sensitivity was 97 and 87%, respectively [103]. Moreover, the reflectance spectrum at 780 nm was found to be moderate in areas of carcinoma in situ, but absent in normal epithelium. The optical properties showed significant changes-more than 80% of the invasive cancer and more than 30% of carcinoma in situ [103]. The group has also found that this modality is suitable for discriminating benign from malignant oral lesions since the reflectance intensity increased as the dysplastic changes increased [104]. Thus, the group has demonstrated that diffusion reflection imaging is a promising technique for the screening of malignant oral lesions and detecting residual disease during operation.

#### Quantum dots imaging

Quantum dots are nanometer-sized semiconductor crystals that luminesce through quantum confinement effects [105, 106]. Quantum dots have several advantages that could overcome the limitations of conventional fluorescent dyes, such as size-tunable emission, wide excitation spectra, strong luminescence and excellent stability against photobleaching [106–108]. In addition, changing the size and composition of quantum dots allows for obtaining a wide range of spectrum, from ultraviolet to the near infrared [109, 110].

**Fig. 5:**
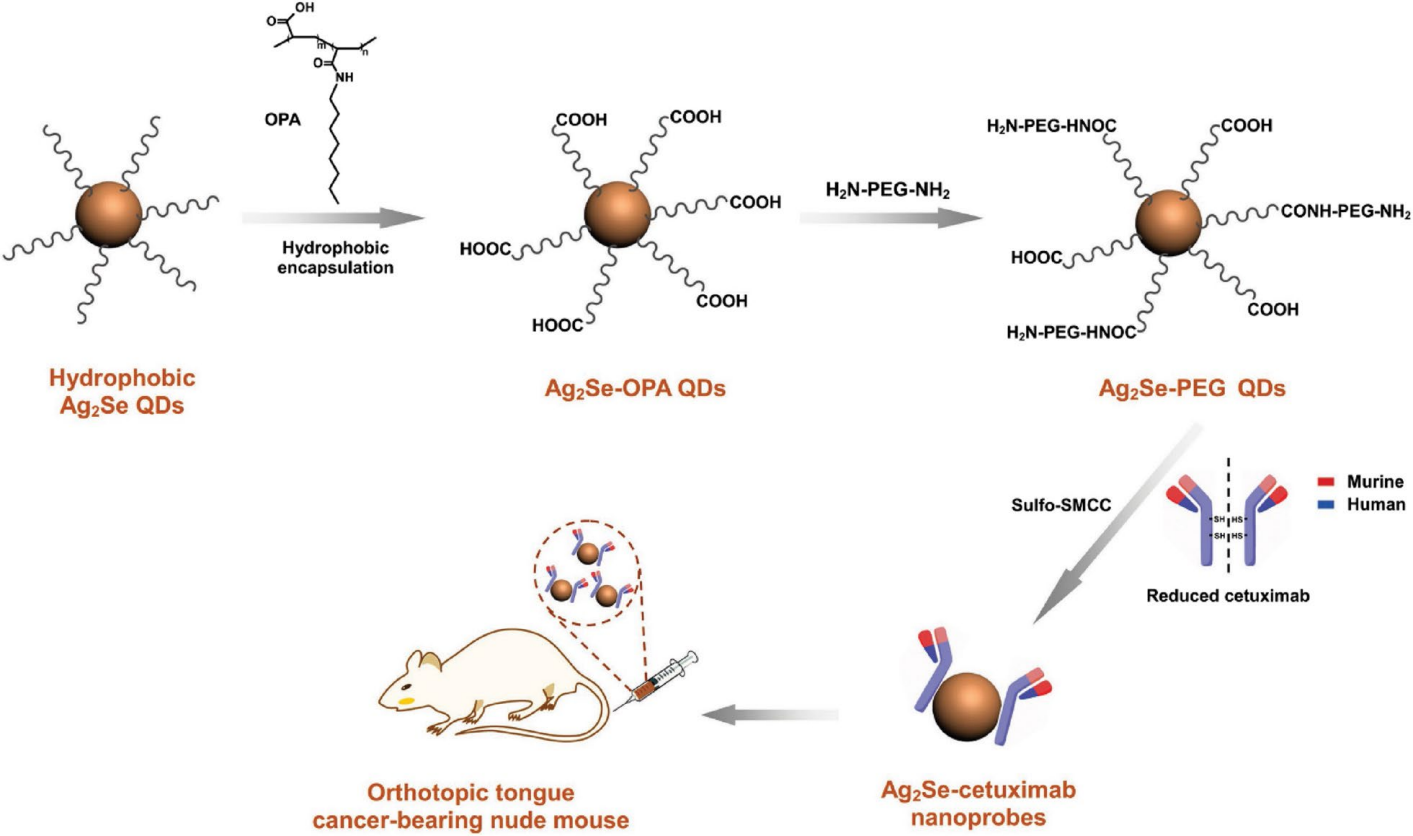
Schematic illustration of surface modification, bioconjugation, and theranostic application of Ag_2_Se QDs coupled with cetuximab (Reprinted with permission from [117]. Copyright 2017 Small)

Currently, quantum dots have been applied in the molecular and cell imaging of OSCC both in vitro and in vivo. It has been demonstrated that quantum dots have high fluorescence intensity, low nonspecific binding, and good stability against photobleaching for the in vitro imaging of human oral cancer cells Tca8113, SCC-25 and BcaCD885 [111–114]. Most of the quantum dots used for in vivo imaging were linked to molecules with the ability to target cancer cells [115]. Recently, it was reported that the near-infrared quantum dots with an emission wavelengths range of 700–900 nm have strong tissue penetration and are not harmful in vivo [114, 115]. Meanwhile, quantum dots with emission wavelengths between 400 and 600 nm are able to avoid the interference of tissue autofluorescence, making them suitable for bioimaging [116, 117]. Studies have proven that quantum dots with an emission wavelength of 800 nm conjugated with EGFR monoclonal antibodies or arginine–glycine–aspartic acid sequence can generate high quality images of OSCC (Fig. 5) [117–119]. The technique also offers great potential in personalized therapy for OSCC [117–119].

### Nano-based ultrasensitive biomarker detection

Currently, plenty of novel proteomic, genomic, and transcriptomic biomarkers are being researched. Exploration of tumor molecular biomarkers-such as tumor necrosis factor-alpha (TNF-α), vascular endothelial growth factor (VEGF), EGFR, and interleukin 6 (IL 6)-holds great promise for early cancer detection and diagnosis [22, 120, 121]. Routine measurement methods-including enzyme-linked immunosorbent assay (ELISA), immunohistochemistry, Western Blot, and polymerase chain reaction-still bear a limited detection sensitivity ranging from pM to fM (10^−12^ to 10^−15^ M) concentration levels [22, 23, 35]. The application of nanotechnology may enhance the detection sensitivity for biomarkers with low concentrations in the tissue samples or body fluids [122, 123].

The saliva peptide finger print technique is a useful tool for salivary proteomics analysis and can predict potential biomarkers valuable for cancer diagnosis [124]. A study utilized matrix-assisted laser-desorption ionization-time-of-flight mass spectrometry (MALDI-TOF–MS) for analyzing the expression spectrum of salivary peptides in 40 OSCC patients and 23 normal controls [125]. Nanomaterial-based magnetic beads were used for selective enrichment of low-molecular-mass peptides. It is noteworthy that 50 proteins expression levels were significantly different between OSCC patients and healthy controls. As a result, the mass peaks of 1285.6 and 1432.2 Da, which were both identified as histatin-3, were correlated with OSCC progression. This study introduced a novel high-throughput, non-invasive strategy for valuable oral cancer biomarkers screening [125]. The specific advantages of magnetic beads constructed on nanomaterial over other types of separation beads have not yet been illustrated.

A nano-based single biomarker detection method has also been utilized for oral cancer detection. A study detected TNF-α by gold protein chip method using a total internal reflection fluorescence microscopy (TIRFM) [35]. A 4 × 5 nanoarray incorporating 500 nm diameter gold spots was achieved on 10 mm square glass substrates. The TNF-α detection sensitivity was reported to be at the attomolar (aM) concentration level (× 10^−18^), enabling ultra-sensitive oral cancer detection [35]. However, this method could not be used for precise quantitative analysis. Another study described the analysis of oral cancer bio marker EGFR with exfoliative cytology specimens of 41 OPMD or OSCC patients and 11 healthy volunteers, using a nano-bio-chip sensor technique [126]. A total of 51 measurement parameters were collected, and biochemical and morphologic changes were further analyzed. The EGFR expression level-along with nuclear area, nuclear diameter, and nuclear-to-cytoplasmic ratio-was significantly altered in oral lesions with diagnosed squamous cell carcinoma or dysplasia [126]. Using ultra-sensitive atomic force microscopy (AFM) and field emission scanning electron microscopy (FESEM) with high resolution (~ 1 nm), another study exhibited the substructure of single human saliva exosomes and interpreted the nanoscale structures of exosomes under varying forces, revealing reversible mechanical deformation [127]. Further, cell-type specific marker CD63 was detected by using 10 nm gold beads on individual exosomes. The nanoscale biomechanical, morphological, and surface biomolecular properties of saliva exosomes are found to be critical for the oral cancer diagnosis [127]. Although these two systems have made it possible for the quantitative analysis of cellular biomarkers, the systems described above can only be used for single biomarker analysis.

It is well-known that single oral cancer biomarkers cannot provide reliable diagnoses [128]. Multiplexed biomarker detection can minimize false positives and negatives arising from single biomarker analysis [128]. A multiplexed biomarker detection approach measured a four-protein panel of biomarkers using an ultrasensitive electrochemical microfluidic array [129]. The microfluidic device contained an array of nanostructured sensors, and plenty of magnetic beads were labeled. The four-protein panel-including interleukin-6, interleukin-8, VEGF, and VEGF-C-was analyzed in 78 oral cancer patient serum samples and 49 controls, and showed a clinical diagnostic sensitivity and specificity for 89 and 98%, respectively [129]. The study provided a low-cost, easily fabricated method for accurate clinical oral cancer diagnosis. Another study analyzed proteins biomarkers in conditioned media of oral squamous cell lines HN12, HN13, OSCC-3 and CAL27 by utilizing a nano ultra-performance liquid chromatography (nano-UPLC) ion-mobility mass spectrometry [130]. A total of approximately 952 proteins-including known cancer biomarker proteins IL-6, IL-8, VEGF-A, and VEGF-C were identified. This nano-UPLC-Q-TOF assay provided a high-throughput approach to quantify proteins and compare protein expression levels across different samples, without the need for stable isotope labeling. The identification of peptides was unlimited with the fragmentation technique [130].

## Conclusion and perspective

**Fig. 6:**
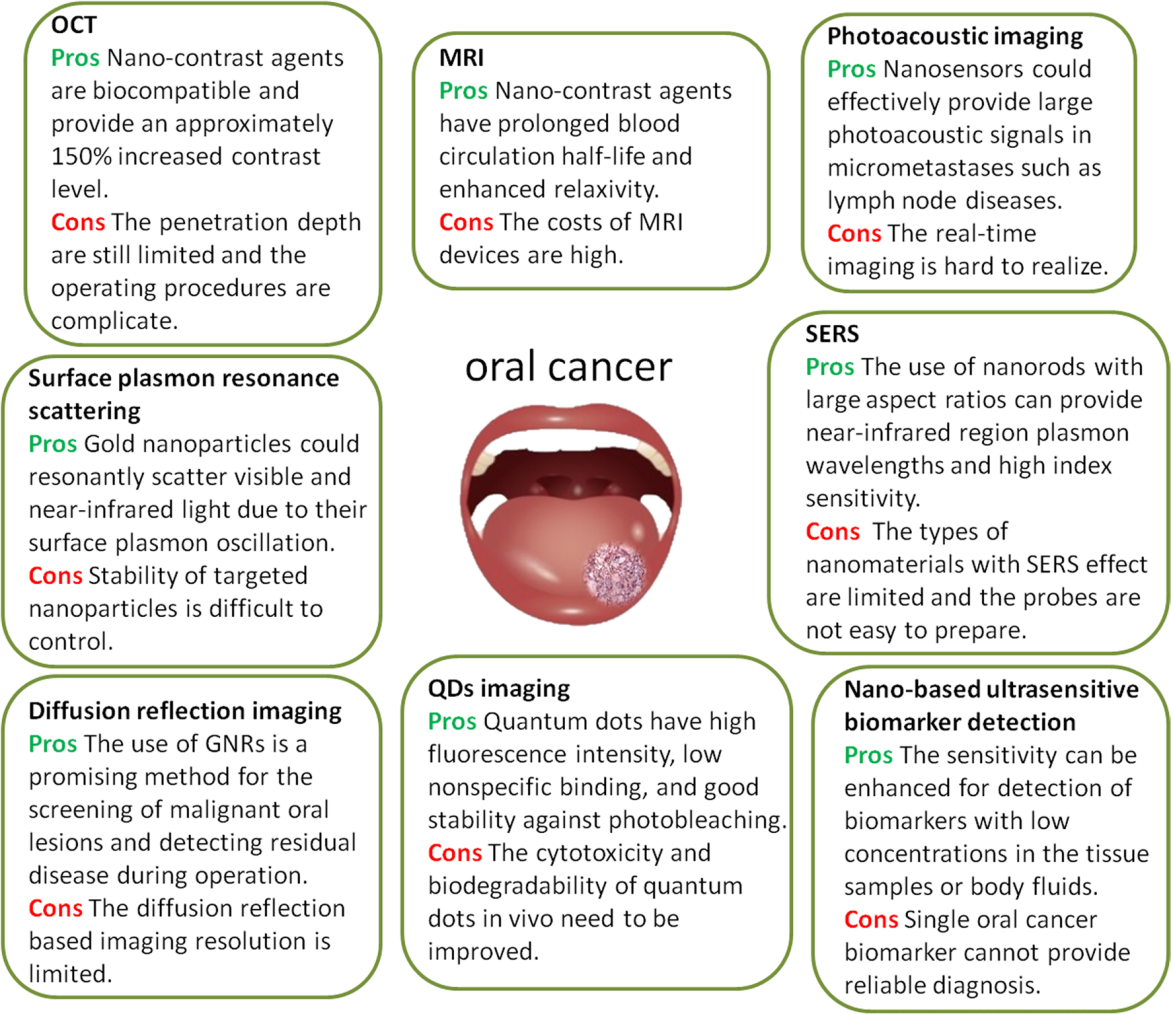
The pros and cons of different nanotechnology for bioimaging and biomarker detection of oral cancer

Ranking as one of the top 10 cancers worldwide, oral cancer has a poor prognosis and a high recurrence rate, and the time and accuracy of diagnosis directly affects disease outcomes [131]. In the past few decades, nanotechnology has brought new techniques to cancer diagnosis [36, 38, 132, 133]. The performance parameters of nanoparticles-such as biocompatibility, function-specific size and shape, blood circulation half-life, and targeting to specific cell surface molecules-can be controlled by modulating their fabrication materials, methods or surface chemistry, making nanoparticles a promising diagnostic material [79]. The present review article has critically introduced nano-based detection strategies for oral cancers, and summarized various kinds of nanomaterials, sample types, and the characteristic of each technique in Table 1. The pros and cons of each nanotechnology for bioimaging and molecular detection of oral cancer were shown in Fig. 6. In the oral cavity, the use of nanoparticles has not only achieved noninvasive real-time diagnosis with high sensitivity and specificity but also assisted with accurately identifying surgical margins, indicating the potential to reduce the reliance on tissue biopsy and histopathological assessment in many cases.

**Table 1:** Summary of nanotechnology based methods for oral cancer detection and diagnosis

Detection method	Nanomaterial type	Surface functionalization	Cell line/sample/model	Characteristic	References
Magnetic resonance imaging	Magnetic PLGA nanoparticles	Surface modified with folate-chitosan conjugate ‘shell’	Prostatic cancer PC3 cells, oral cancer KB cells and normal L929 cells	Shorten the overall T2 relaxation time thereby enhancing the nanoparticle relaxivity to provide better in vitro MR imaging	[59]
	Gd3+ doped amorphous TiO2 nanoparticles	Conjugated with folic acid	HUVEC, PBMC, oral cancer KB cells and normal L929 cells	Enhance image contrast and agent biocompatibility for molecular receptor targeted MRI	[60]
Photoacoustic imaging	Plasmonic nanosensors	Directional conjugated with anti-EGFR monoclonal antibodies and PEG	A metastatic murine model of OSCC	Offer a rapid and effective tool to noninvasively identify micrometastases	[77]
Optical coherence tomography	Spherical Au nanoparticles	Conjugated with anti-EGFR monoclonal antibodies and PEG	A standard hamster cheek pouch model	Enhance the contrast and penetration depth in vivo OCT images	[65]
Surface plasmon resonance scattering	Colloidal gold nanoparticles	Unconjugated or conjugated with anti-EGFR monoclonal antibodies	Nonmalignant epithelial cell line HaCaT, and two malignant oral epithelial cell lines HOC313 clone 8 and HSC3	Find specific molecular biosensor techniques for the diagnosis of oral epithelial living cancer cells in vivo and in vitro	[81]
Surface-enhanced raman spectroscopy	Colloidal gold nanoparticles, self-assembled SERS-active gold nanoparticle monolayer film	Colloidal gold nanoparticles was conjugated with anti-EGFR monoclonal antibodies	Saliva samples from 5 oral cancer patients and 5 healthy individuals	Develop a simple and cost-effective method for preparing highly sensitive SERS-based saliva assay	[63]
	Small spherical gold nanoparticles	Modified with a specific spacer DNA sequence in the core	Oral cancer HSC-3 cells	Improve the current temporal resolution and image quality of Raman-based cell images	[89]
	Plasmonic GNRs	Absorbed on a piece of filter paper	OSCC cell line CAL27, exfoliated cells from 10 healthy individuals and 10 oral cancer patients	Enable highly sensitive, specific, rapid, and noninvasive cancer screening	[94]
Near-infrared absorption imaging	GNRs	Conjugated with Rose Bengal	Human OSCC cell line CAL27 and Tca8113	Demonstrate multi-channel, rapid and quantitative detection of oral cancer cells based on near-infrared absorption	[93]
Diffusion reflection imaging	GNRs	Conjugated with anti-EGFR monoclonal antibodies	A tissue sample of OSCC	Map tumor margins in OSCC with high resolution and depth of penetration	[102]
	GNRs	Conjugated with anti-EGFR monoclonal antibodies	A rat model of OSCC	Introduce a new and simple tool for detecting residual disease intraoperatively	[103]
	GNRs	Conjugated with anti-EGFR monoclonal antibodies	Tissue samples from 15 various dysplastic lesions, 10 OSCC lesions, and 5 healthy controls	Discriminate benign from malignant oral lesions with an objective GNRs reflection measurement	[104]
Quantum dots imaging	Water-soluble quantum dots	Conjugated with biotin and PEG	Human tongue cancer cells Tca8113	Develop of a kind of water-soluble quantum dot for immunofluorescent labeling of cancer cells	[111]
	Goat anti-rabbit QD_655_nm-IgG	QD-IgG compound that binds to survivin and HSP70 by antigen–antibody reaction	Human tongue cancer cells Tca8113	Evaluate the application of quantum dotsand the FITC labeling technique in Tca8113 cells, and to compare the fluorescence intensity and photostability of these techniques	[112]
	Goat anti-mouse QD_525_nm-IgG and goat anti-mouse QD_655_nm-IgG)	QD-IgG compound that binds to HSP70 and HSF-1 by antigen–antibody reaction	Human tongue cancer cells SCC-25	Develop a quantum dot-based approach for heat shock protein 70 and heat shock factor 1 kinetics following heat shock	[113]
	Near-infrared quantum dots	Conjugated with membrane-penetrating polypeptides	Human oral squamous carcinoma BcaCD885 cells	Explore the competence of near-infrared luminescent quantum dots for visual in vivo imaging on oral squamous carcinoma BcaCD885 cells	[114]
	Near-infrared quantum dots	Conjugated with arginine–glycine–aspartic acid	Nude mice bearing head and neck squamous cell carcinoma	Use intravenously injected near-infrared quantum dots conjugated with arginine-glycine-aspartic acid to generate high quality images of head and neck squamous cell carcinoma	[118]
	Near-infrared quantum dots	Conjugated with anti-EGFR monoclonal antibodies	OSCC nude mice model	Investigate in vivo visible imaging of OSCC by targeting EGFR with near-infrared quantum dots	[119]
	Near-infrared quantum dots	Conjugated with anti-EGFR monoclonal antibodies	Orthotopic tongue cancer-bearing nude mice	Construct multifunctional Ag_2_Se–cetuximab quantum dots for targeted imaging and therapy of orthotopic tongue cancer	[117]
Saliva peptide finger print analysis	Nano magnetic beads	Have a magnetic core enabling weak cation exchange	Whole saliva samples from 40 OSCC patients and 23 healthy controls	Predict potential biomarkers for OSCC diagnosis	[125]
Single biomarker detection	Gold nanoarray	Binded to the Fc region of the TNF-α capture antibody	Samples (type unknown) from an OSCC patient	Enable ultrasensitive detection of TNF-α	[35]
	Nano-bio-chip	Labeled with anti-EGFR monoclonal antibodies	Brush biopsy from 41 OPMD or OSCC patients and 11 healthy volunteers	Provide rapid detection and quantitation of EGFR biomarker	[126]
	Gold nano beads	Coated with antiCD63 IgG secondary antibody	Saliva samples from healthy volunteers	Explore quantitative approaches to biochemical characterization of exosomes	[127]
Multiplexed biomarker detection	Nanostructured microfluidic array	Combined gold nanoparticle surfaces with magnetic beads massively labeled with horseradish peroxidase enzyme labels	78 serum samples from oral cancer patients and 49 cancer-free controls	Provide a rapid four-protein panel serum test	[129]
	Nano-UPLC	Label-free	Squamous cancer lines HN12, HN13, OSCC-3, CAL27 and normal epidermal keratinocyte noncancer line HaCaT	Develop a lable-free approach to identify and quantify proteins in complex samples	[130]

*PLGA* poly lactide-co-glycolide, *OSCC* oral squamous cell carcinoma, *TNF*-*α* tumor necrosis factor-alpha, *EGFR* epidermal growth factor receptor, *PEG* polyethylene glycol, *UPLC* ultra-performance liquid chromatography, *GNRs* gold nanorods, *HUVEC* human primary endothelial cells, *PBMC* peripheral blood mononuclear cells, *OCT* optical coherence tomography

Nano-based contrast agents for MRI, OCT and photoacoustic imaging have lower toxicity, prolonged blood circulation half-life, and the ability to target unique cell surface molecules. Compared to routine contrast agents, nano agents exhibit better image contrast properties and improved penetration depth. In optical imaging, nanoparticles enable sufficient signals and sub-cellular spatial resolution. They can generate surface plasmon resonance at near-infrared wavelengths, gathered around the targeted cell surface, and the optical resonance properties of nanorods can be regulated over a broad range by adjusting their sizes and shapes. Quantum dots with size-tunable emission, wide excitation spectrum, high intensity of luminescence, and excellent photochemical stability have overcome the disadvantages of traditional fluorescence markers. As for cancer biomarker detection, nano-based materials-such as nano beads, gold nanoarray, and nano-bio-chips-offer high throughput screening for potential biomarkers and have brought the level of detection sensitivity to the nanoscale. Therefore, the small and earlier intraepithelial lesions missed by common techniques can potentially be detected by nanotechnologies, making oral diseases more readily cured.

Nano-based diagnostic methods act as a promising tool to provide real-time, convenient, and cost-effective diagnosis for oral cancer detection and diagnosis. They can provide molecular targeted imaging, analyze biomarkers at nano-scale, enable intraoperative identification of surgical resection margins, and monitor oral cancer prognosis after treatment. Although these technologies have been studied in ex vivo studies of tissue and saliva samples and in vivo studies in animal models, further efforts should be employed before these strategies can be successfully applied in clinical diagnosis.

## Abbreviations

OPMD: oral potentially malignant disorders
OSCC: oral squamous cell carcinomas
TB: toluidine blue
EGFR: epidermal growth factor receptor
MRI: magnetic resonance imaging
CT: computed tomography
CBCT: cone beam computed tomography
PET: positron emission tomography
Gd-DTPA: Gd3+ complexed with diethyltriamine-pentaacetic acid
Gd-DOTA: tetra azacyclododecane-1,4,7,10-tetraacetic acid
SPIO: superparamagnetic iron oxide
USPIO: ultrasmall superparamagnetic iron oxide
MAPS: molecularly activated plasmonic nanosensors
SERS: surface-enhanced Raman scattering
GNRs: gold nanorods
LSPR: longitudinal surface plasmon resonance
RB: rose bengal
TNF-α: tumor necrosis factor-alpha
VEGF: vascular endothelial growth factor
IL 6: interleukin 6
ELISA: en-zyme-linked immunosorbent assay
MALDI-TOF–MS: matrix-assisted laser-desorption ionization-time-of-flight mass spectrometry
TIRFM: total internal reflection fluorescence microscopy
AFM: atomic force microscopy
FESEM: field emission scanning electron microscopy
PEG: polyethylene glycol
UPLC: ultra-performance liquid chromatography
PLGA: poly lactide-*co*-glycolide
HUVEC: human primary endothelial cells
PBMC: peripheral blood mononuclear cells
